# Supplementary material for: TimelinePTC: Development of a unified interface for pathways to care collection, visualization, and collaboration in first episode psychosis
Source: PLoS One. 2024 Jul 19;19(7):e0302116. doi: 10.1371/journal.pone.0302116 (PMC11259254; doi:10.1371/journal.pone.0302116)
Supplement: S1 Table — (DOCX) [file pone.0302116.s001.docx]

[Framing]

Let us now try to look at the pathways to care. Individuals and families often follow unique pathways to access care, when seeking help after the onset of illness (psychosis). We are interested in learning about your journey since your symptoms first appeared and try to capture each help-seeking event. During our conversation, we will attempt to recall and record the date of every interaction – both clinical and non-clinical – that occurred along the path. When exact dates are not available, we will do our best to estimate. We will enter this information in a visual tool where you can see this information and fill in the gaps, if any.

[Onset] <- to be resolved with SIPS onset date later. [*Structured Interview for Psychosis Risk Syndromes -- a clinical assessment tool used to identify and diagnose psychosis risk symptoms. It is employed by STEP to determine the presence and severity of psychotic symptoms, guiding appropriate treatment and intervention strategies.*]

Your best guess, approximately when did you or others report noticing a difference in your thoughts or behaviors? Going forward we will use the phrase “these experiences” to refer to these changes.

[Clinical Nodes]

[ED] Between then and now, have you been to an Emergency Department over concerns about these experiences?

If so, tell me when did this take place?

Who was involved in you going there? For example, did you take yourself? Was a family member or teacher involved? Were police involved? Did another clinical provider suggest it? Or someone else – or multiple others – we haven’t mentioned?

And what happened after that?

[Inpt] Have you been admitted to the hospital (inpatient) over concerns about these experiences?

If so, tell me when did this take place?

Who was involved in you going there?

And what happened after that?

[IOP] Have you been engaged with an intensive outpatient program (IOP) as part of your treatment? [*An intensive outpatient program (IOP) is* *a structured, non-residential treatment program for individuals with mental health or substance use disorders. It provides therapy and support multiple times a week for several hours each day, allowing participants to continue with their daily lives while receiving intensive treatment. IOPs are designed to offer a higher level of care than traditional outpatient therapy but are less intensive than inpatient programs, making them suitable for individuals who need significant support but do not require 24-hour supervision.*]

If so, tell me when did this take place?

Who was involved in you going there?

And what happened after that?

[PCP] Has your primary care provider ever discussed these experiences with you, attempt to treat them, or refer you to treatment somewhere else?

If so, tell me when did this take place?

Who was involved in you going there?

And what happened after that?

[Outpt] Have you been treated by an outpatient mental health provider for these experiences?

If so, tell me when did this take place?

Who was involved in you going there.

And what happened after that?

[Acute] Have you been treated evaluated by an acute walk-in mental health service over concerns about these experiences? [*An acute walk-in mental health service is a healthcare facility that provides immediate, on-demand mental health care for individuals experiencing severe or crisis-level mental health issues. These services offer rapid assessment, intervention, and support without the need for an appointment, helping to stabilize patients and direct them to appropriate ongoing care or treatment as needed. They serve as an alternative to emergency room visits for urgent mental health needs.*]

If so, tell me when did this take place?

Who was involved in you going there.

And what happened after that?

[Mobile] Have you been evaluated by a team who came to you in your home or the community over concerns about these experiences?

If so, tell me when did this take place?

Who was involved in you going there?

And what happened after that?

[OtherMH] Have you been treated by any other outpatient mental health provider – substance use treatment, home-based care, prison-based care?

If so, tell me when did this take place?

Who was involved in you going there?

And what happened after that?

[OtherMed] Have other medical providers – not mental health and not primary care providers such as inpatient medical providers ever discussed these experiences with you, attempt to treat them, or refer you to treatment somewhere else?

If so, tell me when did this take place?

Who was involved in you going there?

And what happened after that?

[Other] Were there other clinical professionals who discussed these experiences with you that we have not mentioned yet?

If so, tell me when did this take place?

Who was involved in you going there?

And what happened after that?

[AP] When, if ever, did you first take a [antipsychotic] medication to help with these experiences?

[Community fill-in] Were there times that you, your family, friends, police, counselors, teachers, or others (community nodes) expressed concern or attempted to get you into care for these experiences but were not able to?

If so, tell me who was involved in you going there.

And what happened after that?

[Review] Let us look back at the data we have collected, looking carefully at the interactions we have recorded, including the label and date of each. Are there any we left out? On days when multiple interactions happened, are the interactions in the correct order?
