# Supplementary material for: TimelinePTC: Development of a unified interface for pathways to care collection, visualization, and collaboration in first episode psychosis
Source: PLoS One. 2024 Jul 19;19(7):e0302116. doi: 10.1371/journal.pone.0302116 (PMC11259254; doi:10.1371/journal.pone.0302116)
Supplement: S2 Table — (DOCX) [file pone.0302116.s002.docx]

|  |  | **Total**  **Encounters**  (n = 1,117) | | **Unique**  **participant**  **encounters** | | **Demand**  **encounters** | | **Marginal-delay per**  **demand encounter**  (days) | | **Supply**  **encounters** | | **Marginal-delay per**  **supply encounter**  (days) | |
| --- | --- | --- | --- | --- | --- | --- | --- | --- | --- | --- | --- | --- | --- |
|  |  |  |  | (n = 156) | |  | | Median | Range |  | | Median | Range |
| **Community** | **Family** | 198 | 17.7% | 121 | 77.6% | 140 | 44.2% | 0 | 0-519 | 58 | 42% | 0 | 0-126 |
|  | **Self** | 121 | 10.8% | 62 | 40% | 72 | 23% | 0 | 0-61 | 49 | 35% | 0 | 0-14 |
|  | **Police** | 84 | 7.5% | 63 | 40% | 63 | 20% | 0 | 0-149 | 21 | 15% | 0 | 0-30 |
|  | **Other** | 41 | 3.7% | 31 | 20% | 32 | 10% | 0 | 0-388 | 9 | 6% | 0 | 0-24 |
|  | **Education** | 12 | 1.1% | 10 | 6.4% | 10 | 3.2% | 0 | 0-954 | 2 | 1% | 8.5 | 0-17 |
|  | **Total** | 456 | 40.8% |  |  | 317 |  |  |  | 139 |  |  |  |
| **Clinical** | **ED** | 255 | 22.78 | 137 | 87.8% | 131 | 57.5% | 0 | 0-69 | 124 | 28.6% | 0 | 0-187 |
|  | **Inpt** | 191 | 17.1% | 122 | 78.2% | 25 | 11% | 12 | 3-335 | 166 | 38.3% | 13 | 0-820 |
|  | **Outpt** | 101 | 9.0% | 65 | 42% | 30 | 13% | 36.5 | 0-584 | 71 | 16% | 56 | 0-724 |
|  | **IOP** | 44 | 3.9% | 28 | 18% | 2 | 0.9% | 19.5 | 0-39 | 42 | 9.7% | 29 | 1-896 |
|  | **Acute** | 30 | 2.7% | 26 | 17% | 14 | 6.1% | 5.5 | 0-305 | 16 | 3.7% | 5.5 | 0-333 |
|  | **PCP** | 23 | 2.1% | 19 | 12% | 17 | 7.5% | 2 | 0-354 | 6 | 1% | 1.5 | 0-129 |
|  | **OtherMH** | 7 | 0.6% | 4 | 3% | 3 | 1% | 0 | 0-0 | 4 | 1% | 107.5 | 21-212 |
|  | **Mobile** | 6 | 0.5% | 6 | 4% | 5 | 2% | 0 | 0-1 | 1 | 0.2% | 0 | 0-0 |
|  | **OtherMed** | 4 | 0.4% | 4 | 3% | 1 | 0.4% | 109 | -- | 3 | 1% | 41 | 8-150 |
|  | **Total** | 661 | 59.2% |  |  | 228 |  |  |  | 433 |  |  |  |
