# Supplementary material for: TimelinePTC: Development of a unified interface for pathways to care collection, visualization, and collaboration in first episode psychosis
Source: PLoS One. 2024 Jul 19;19(7):e0302116. doi: 10.1371/journal.pone.0302116 (PMC11259254; doi:10.1371/journal.pone.0302116)
Supplement: Attachment — Submitted filename: TimelinePTC_PlosOne.docx [file pone.0302116.s003.docx]

TimelinePTC: Development of a unified interface for pathways to care collection, visualization, and collaboration in first episode psychosis

This is an interesting study that describes a tool that has been developed to easily visualize pathways to care in first episode psychosis patients. It highlights how an original tool has been modified to make it more efficient in data capture and data visualization.

Two issues that need resolving.

1. The paper has been written assuming that the reader knows a lot about specialized treatment early in psychosis programs. Therefore, the supplementary data include information that is not easy to understand for a non-specialist reader. The tool picks information from various sources that one has to know of to understand what it is doing. For example, it refers to the Structured Interview for Psychosis Risk Syndromes, intensive outpatient programs, mobile community teams etc. These are technical interventions that are not often routine care in psychosis care. There is need to explain these sources of information and what they do.
2. I do not know why the tables and figures are supplementary data. It would be better if they were included in the text. It makes reading and understanding easier.
