# Supplementary material for: TimelinePTC: Development of a unified interface for pathways to care collection, visualization, and collaboration in first episode psychosis
Source: PLoS One. 2024 Jul 19;19(7):e0302116. doi: 10.1371/journal.pone.0302116 (PMC11259254; doi:10.1371/journal.pone.0302116)
Supplement: Attachment — Submitted filename: Response Letter TimelinePTC_PlosOne.docx [file pone.0302116.s004.docx]

Review Comments to the Author

**Reviewer #1**:

TimelinePTC: Development of a unified interface for pathways to care collection, visualization, and collaboration in first episode psychosis

This is an interesting study that describes a tool that has been developed to easily visualize pathways to care in first episode psychosis patients. It highlights how an original tool has been modified to make it more efficient in data capture and data visualization.

**Thank you for your kind words and your thoughtful feedback that greatly improved the manuscript.**

Two issues that need resolving.

1. The paper has been written assuming that the reader knows a lot about specialized treatment early in psychosis programs. Therefore, the supplementary data include information that is not easy to understand for a non-specialist reader. The tool picks information from various sources that one has to know of to understand what it is doing. For example, it refers to the Structured Interview for Psychosis Risk Syndromes, intensive outpatient programs, mobile community teams etc. These are technical interventions that are not often routine care in psychosis care. There is need to explain these sources of information and what they do.

**This is a valid point. We thoroughly reviewed the primary text of the article to make sure any obscure language or jargon were more fully explained. Many of the terms the reviewer mentions (local care modalities – intensive outpatient, mobile evaluation, etc) are again specific to the care network in which the program was developed and, as the manuscript says, these are completely modifiable by editing the open-source software. While the meanings of these exact terms are not inherently germane to the broader description of the software, we have revised the explanatory glossary to caption of the PTC network graph (now Figure 2).**

**Other terms the reviewer mentioned as being not commonly understood (Structured Interview for Psychosis Risk Syndromes) are terms buried deep in the supplementary materials, in the case the semi-structured interview script that the research assistants use to collect PTC data. This script is really for internal use and not part of the research product described by this manuscript – something we included only for illustrative completeness. As a courtesy, we have added some parenthetical notes to some terms to make the script a bit more universally interpretable.**

2. I do not know why the tables and figures are supplementary data. It would be better if they were included in the text. It makes reading and understanding easier.

**We think some of the confusion here is the way the submission is packaged that makes the in-text figures (which are put at the end of the document at this stage of review) and the supplementary materials (which follow right after) difficult to distinguish. We at first only had the screen shots of TimelinePTC in the document, but on the reviewer’s suggestion have moved the PTC network figure into the main body (Fig. 2). We maintain that the interview script and the tabular analysis of PTC data are better left in the supplementary materials.**

**Reviewer #2:**

Would like to see more thoughtful discussion on how piloting and implementation across Epinet Hubs could occur.

**While this was not mentioned in the paper, we have in fact thought of this application and so we added some language to the discussion.**

Nodes related to criminal justice (ieg Jail) could be added.

**The PTC data we collected for STEP did include 3-4 instances of prison-based mental health care. In our data, these were so sparse that they were eventually grouped under “OtherMH” – see expanded glossary of terms under Fig.2. If a particular region or healthcare network found any given “other” node featured often enough, it could be added to the those node labels called out explicitly by editing the source code for the software. This is a great example of the regional-responsivity we mention as both a limitation of the study and a strength of the software project. We have added some language around this to page 6.**

How are psychotropics along the pathway (ie not antipsychotics) considered?

**We added some language to defend this decision on page 6.**

How are the reasons for the encounter captured?

**This is an insightful question. We have added some language on page 6 to address this, primarily referencing the careful framing of the interview script.**
